# Novel strategy for manufacturing autologous dendritic cell/allogeneic tumor lysate vaccines for glioblastoma

**DOI:** 10.1093/noajnl/vdaa105

**Published:** 2020-08-26

**Authors:** Ian F Parney, Michael P Gustafson, Mary Solseth, Peggy Bulur, Timothy E Peterson, James B Smadbeck, Sarah H Johnson, Stephen J Murphy, George Vasmatzis, Allan B Dietz

**Affiliations:** 1 Department of Neurological Surgery, Mayo Clinic, Rochester, Minnesota, USA; 2 Department of Immunology, Mayo Clinic, Rochester, Minnesota, USA; 3 Human Cell Therapy Lab, Mayo Clinic, Rochester, Minnesota, USA; 4 Division of Transfusion Medicine, Department of Laboratory Medicine and Pathology, Mayo Clinic, Rochester, Minnesota, USA; 5 Division of Genetics and Bioinformatics, Mayo Clinic, Rochester, Minnesota, USA; 6 Center for Individualized Medicine, Mayo Clinic, Rochester, Minnesota, USA

**Keywords:** dendritic cell, glioblastoma, manufacturing, vaccine

## Abstract

**Background:**

Glioblastoma, the most common primary malignant brain tumor, is nearly universally fatal by 5 years. Dendritic cell vaccines are promising but often limited clinically by antigen choice, dendritic cell potency, and/or manufacturing yield. We optimized vaccine manufacture, generating potent mature autologous dendritic cells pulsed with allogeneic glioblastoma lysates.

**Methods:**

Platelet lysate-based supplement was used to establish human glioblastoma cell lines. Phenotype and genotype were assessed. An improved culture technique to generate mature dendritic cells from glioblastoma patients’ monocytes was developed. The ability of T cells stimulated with autologous dendritic cells pulsed with allogeneic glioblastoma cell lysate to kill HLA-A2-matched glioblastoma cells was assessed.

**Results:**

Glioblastoma cell lines established with platelet lysate supplement grew faster and expressed more stem-like markers than lines grown in neural stem cell media or in the presence of serum. They expressed a variety of glioma-associated antigens and had genomic abnormalities characteristic of glioblastoma stable up to 15 doublings. Unlike standard culture techniques, our optimized technique produced high levels of mature dendritic cells from glioblastoma patients’ monocytes. Autologous T cells stimulated with mature dendritic cells pulsed with allogeneic glioblastoma cell line lysate briskly killed HLA-A2-matched glioblastoma cells.

**Conclusions:**

Our glioblastoma culture method provides a renewable source for a broad spectrum glioblastoma neoantigens while our dendritic cell culture technique results in more mature dendritic cells in glioblastoma patients than standard techniques. This broadly applicable strategy could be easily integrated into patient care.

**Key Points**

- Current dendritic cell (DC) vaccine manufacture is not ideal due to the need for fresh tumor and reliance on immature DCs.
- Glioblastoma (GBM) cell lines with platelet lysate supplement provide a diverse renewable antigen source.
- Culture techniques must be optimized with GBM patients’ monocytes to generate mature DCs.

**Importance of the Study**

Immunotherapy is revolutionizing cancer treatment but results for glioblastoma (GBM) have lagged behind. Dendritic cell (DC) vaccines have been promising experimentally for nearly 2 decades but have yet to be widely adopted. Most GBM DC vaccines use individual patients’ fresh tumor as an antigen source. This limited resource does not facilitate antigen-specific response testing. Furthermore, most GBM DC vaccines use culture techniques without maturation steps or maturation steps optimized with healthy donor monocytes that are not effective for GBM patients due to enrichment with immunosuppressive cells. Only mature DCs are fully immunostimulatory while immature DCs induce T-cell anergy. We report important progress addressing these issues. A bank of clinical-grade human GBM cell lines was established with novel technology as a renewable, diverse antigen source. Culture techniques were optimized to generate mature DCs from GBM patients’ monocytes. These manufacturing advances yield a potent and widely applicable GBM DC vaccine.

Glioblastoma (GBM) is the most common primary malignant brain tumor. Median overall survival (OS) with surgical resection, radiation, and temozolomide chemotherapy is 14.6 months.^1^ GBM vaccines have been promising in small clinical trials^2–4^ but have demonstrated less efficacy^5^ or been subject to large-scale dropout between screening and treatment due to a variety of reasons^6,7^ in larger, randomized studies. In part, this may reflect antigen choice, vaccine potency, and/or manufacturing obstacles.

Many GBM vaccine strategies have focused on bulk antigens derived from patients’ tumors.^2,4^ While highly personalized and providing a large library of antigens, fresh tumor tissue is a limited resource that is not always accessible. Even when available, it can be challenging to generate enough vaccines to administer more than a few doses and antigen-specific response testing is not possible as every patient’s vaccine contains different antigens. Alternatively, some vaccines rely on specific antigen(s) known to be expressed by some GBMs.^3,5,8,9^ This facilitates vaccine production and antigen-specific response testing. However, many peptide vaccines in this category are limited to specific human leukocyte antigen (HLA) haplotypes^8,9^ and targeting a handful of antigens provides an inherent pathway for treatment resistance through immunoediting or loss of expression of these antigens by the tumor.^3,5^

Dendritic cell (DC) vaccines are an attractive platform for cancer immunotherapy.^10^ DCs are potent antigen-presenting cells critical to initiating adaptive immune responses. They are generated from CD14+ monocytes in vitro through a series of culture steps to yield immature DCs followed by mature DCs. This last transition (accompanied by CD83 upregulation) is key as only mature DCs are fully immunostimulatory.^11,12^ Immature DCs may actually induce T-cell anergy following antigen presentation in the absence of appropriate co-stimulation.^13^ Most culture techniques generating DCs for cancer vaccine trials have been optimized using healthy donor monocytes.^14,15^ Unfortunately, cancer patients (including GBM patients) have circulating monocyte populations that are enriched for immunosuppressive variants such as myeloid-derived suppressor cells.^16,17^ It is not clear that standard culture techniques generate similar mature DC yields from GBM patients’ monocytes.

To address these issues, we developed a novel autologous mature DC/allogeneic GBM lysate vaccine strategy.

## Methods

### Patient Samples

All blood and GBM tissue samples were obtained intraoperatively in adult recurrent or biopsy-proven GBM patients undergoing surgery for clinical indications. This was reviewed and approved by the Mayo Clinic Institutional Review Board (IRB#06-002617 and IBR#13-000808). After written, informed consent, patients were tested for eligibility as a tissue donor including completion of a donor questionnaire and infectious disease blood tests to confirm eligibility as a cGMP tissue donor (cGMP; current Good Manufacturing Practices; guidelines issued by the FDA required to allow tissues and cells to be used for clinical therapy). Patients with positive responses to this questionnaire were excluded. Healthy volunteer peripheral blood mononuclear cells were obtained from discarded anonymous leukoreduction system chambers processed in the Mayo Clinic Blood Bank.^18^

### Establishing cGMP Human GBM Cell Lines

Patients underwent craniotomy and resection with a single surgeon (I.F.P.). After intraoperative pathological confirmation of malignant glioma, staff from the Mayo Immune Progenitor and Cell Therapy (IMPACT) Laboratory transported fresh, sterile GBM tissue from the OR to the laboratory for manufacturing. Tumor tissue was processed via a BD Medimachine and equivalent volumes of digested tumor placed into a culture into 1 of 3 media. Mayo cGMP media (AIM-V; Thermo-Fisher; 5% PLTMax; Mill Creek Life Sciences; 2 U/mL heparin; APP Pharmaceuticals; and Pen/Strep) contain cGMP-compatible human platelet lysate (PLTMax) as a key supplement that contains a natural repair proteome supporting the growth of many cell types.^19^ Neural Stem Cell (NSC) media contains Neurobasal-A media with N2 and B27 (void of vitamin A) supplements (Invitrogen), 50 ng/mL recombinant EGF and FGF (R&D Systems), antibiotic (Pen/Strep), and glutamine (Invitrogen). Standard fetal bovine serum (FBS)-based media are DMEM/F12 (Invitrogen) supplemented with 10% FBS, penicillin/streptomycin, and glutamine.

### Growth and Expression Patterns of cGMP Human GBM Cell Lines

For those lines with sufficient growth in each condition to allow, cells were used to evaluate the CD133 expression by flow cytometry using standard staining and analysis methods. Immunofluorescence staining was performed for primitive (nestin, SOX2) and mature (GFAP, ephrin A2) glioneuronal markers. Western blot was performed for common GBM-associated antigens (EGFR, EGFR-VIII, Erb-B2, gp100, MAGE-A3, IL13Rα2, and p53) selected based on predetermined criteria^20^ for evaluation in tumor immunotherapy studies. Cultured cells were lysed in buffer containing (in mmol/L) 50 NaCl, 50 NaF, 50 sodium pyrophosphate, 5 EDTA, 5 egtazic acid, and 2 Na_3_VO_4_ and 1% Triton X-100, 0.5 mmol/L phenylmethylsulfonyl fluoride, 10 μg/mL leupeptin, and 10 mmol/L 4-(2-hydroxyethyl)-1-piperazineethanesulfonic acid, pH 7.4. Cell lysates were sonicated for 3 s prior to protein content analysis using the Bradford protein assay (Bio-Rad). Soluble protein extracts (20 μg) were loaded into 12.5% polyacrylamide gels and transferred onto polyvinylidene fluoride membranes. Membranes were incubated (1 h) in a blocking buffer followed by incubation (1 h) with primary antibody. After 1 h incubation with secondary antibody, membranes were visualized by enhanced chemiluminescence (Pierce). The source and clones for all antibodies used in this manuscript are detailed in Supplementary Table 1.

### Genomic Abnormalities and Stability in cGMP Human GBM Cell Lines

Mate-pair sequencing (MPseq) provides a whole-genome-based structural variance analysis within a genome utilizing a specialized library preparation designed to tile the genome with large 2–5 kb genomic fragments.^21–23^ In addition to copy number variation (CNV), MPseq defines breakpoints of discordant genomic junctions from rearrangements that frequently drive tumor phenotypes with specialized bioinformatics that reduces false positives. MPseq enables accurate genome profiling to determine whole and partial chromosome gains/losses, loss of heterozygosity, single and biallelic gene losses, and precise structures of recombinant DNA junctions and the impact on genes, including fusions, truncations, or promoter losses.

One microgram of DNA was applied to MPseq library preparation using the Nextera Mate-Pair Kit (Illumina) following the manufacturer’s instructions. Libraries were sequenced on the Illumina HiSeq4000 platform at a depth of 4 libraries per lane. For MPseq data, the BIMA (binary indexing mapping algorithm), developed by the Biomarker Discovery Lab at Mayo Clinic, simultaneously maps both reads in a fragment to the GRCh38 reference genome.^24^ Structural variants were detected using SVAtools, a suite of algorithms also developed by the Biomarker Discovery Lab at Mayo Clinic.^21^ SVAtools specifically detects discordant fragments supporting a common junction (supporting fragments) with powerful masks and filters to remove false-positive junctions. CNV detection is performed using the read count of concordant fragments within non-overlapping bins.^23^ This algorithm uses both a sliding window statistical method to determine likely copy number edges from read depth and breakpoint locations determined in the junction detection stage to more accurately place these edges. Once the genome was segmented into likely copy number regions, the normalized read depth for a region was calculated as 2 times the read depth within a region divided by the expected read depth for normal diploid level for the sample. Chromosomal copy levels and discordant mapping junctions are visualized on interactive software for genome plots.^22^  *Z*-scores were calculated from supporting read numbers normalized for overall total fragments yielded from the MPseq. Average and standard deviation for supporting read levels were detected for each junction. *Z*-score was calculated using the formula: [(normalized supporting read value – mean supporting read value)/standard deviation of mean].

### Generating Mature DC

All DC culture starts with either whole blood or fluid bed cell collection. Mononuclear cells were isolated by density centrifugation and CD14+ cells were collected by immunomagnetic selection (Miltenyi Biotec). GM-CSF was either pharmaceutical grade (Leukine; Partner Therapeutics) or R&D Systems. All other cytokines were from R&D Systems. Chemicals were from Sigma. Viable cells were plated on one of the following conditions:

*M1*: RPMI1640 + 10% human AB serum (HABS), GM-CSF (1000 IU/mL), IL-4 (500 IU/mL) for 7 days.

*M2*: RPMI1640 + 2% HABS, GM-CSF (1000 IU/mL), IL-4 (500 IU/mL) for 24 h, then the same plus TNF-a (1100 IU/mL), PGE_2_ (1 µg/mL), IL-1b (10 ng/mL), and IL-6 (10 ng/mL) for 24 h.

*M3*: X-Vivo 15 + 1% HABS, GM-CSF (1000 IU/mL), IL-4 (500 IU/mL) for 3 days, then the same plus TNF-a (1100 IU/mL) and PGE_2_ (1 µg/mL) for 2 days.

*M4*: Sigma Stemline DC media + GM-CSF (1000 IU/mL), IL-4 (500 IU/mL) for 3 days, then the same plus TNF-a (1100 IU/mL) and PGE_2_ (1 µg/mL) for 2 days.

*M5*: DC-OPT1: Sigma Stemline DC media + GM-CSF (1000 IU/mL), IL-4 (500 IU/mL) for 3 days then the same plus TNF-a (1100 IU/mL), PGE_2_ (1 µg/mL), and poly I:C for 2 days.

*M6*: Proprietary

*M7*: Proprietary

Cells were collected and surface marker expression (CD83, CD80, CD86, HLA-DR, and CCR7) was determined by flow cytometry.

### Generating Antigen-Primed DCs

Tumor lysate (TL) was prepared from 2 cGMP human GBM cell lines by sonication and 5 freeze/thaw cycles. DCs generated using method M7 (optimized for generating mature CD83 + DCs from GBM patients’ monocytes) were pulsed with 0.1 mg/mL of TL (approximately 15 mg) and incubated for 18 ± 6 h.

#### In vitro stimulation of T cells with autologous DC or DC-TL

Frozen autologous CD3, DC-TL, and DC were used as source material for this assay. CD3 cells were stimulated with autologous DC-TL (TL-pulsed DC) or DC alone (CD3:DC = 5:1) in AIM-V with 1% HABS, 1% Penicillin/Streptomycin, and 25 ng/mL IL-7. Culture media (1 mL) was removed and replaced with 1 mL AIM-V media supplemented with 2.5% PLTMax on days 3 and 5 of culture plus IL-2 (100 U/mL on day 3 and 50 U/mL on day 5). CD3 cells cultured alone with designated IL-7 and IL-2 cytokines served as controls. On day 7, T cells (CD3-cytokine, CD3-DC, CD3-DCTL) were harvested and replated for a second round of stimulation following the same stimulation procedure. After 14 days in culture, expanded CD3 from each stimulation/expansion condition were harvested and used in CTL killing assay of an HLA-A2+, GFP-labeled Mayo cGMP human GBM cell line.

#### In vitro GBM cell line beta-GFP cytotoxicity assay

Beta-GFP-labeled GBM cells were seeded at 5000 cells per well in 96-well flat-bottom tissue culture plates in AIM-V with 5% PLTMax, 1% GlutaMAX, 1% Penicillin/Streptomycin, and 2 U/mL Heparin. After 24 h incubation for cells to attach, media were removed and 50 000 culture-expanded T cells (CD3-cytokine, CD3:DC/TL, or CD3:DC) in AIM-V media plus 1% HABS in a final volume of 200 μL were layered over the attached GBM cells. Baseline GBM cell count imaging scan was acquired prior to the removal of GBM cell line base media and every 4 h after the addition of T cells using the IncuCyte Zoom (Essen BioScience).

### Statistical Analysis

Where applicable, the Mann–Whitney test, Wilcoxen test, correlation or *T* tests were performed by GraphPad Prism version 7.0e for Mac OSX, GraphPad Software (www.graphpad.com).

## Results

### Mayo cGMP Media Is More Efficient for Establishing/Expanding Human GBM Cell Cultures Than NSC or FBS Media

**Figure 1.**
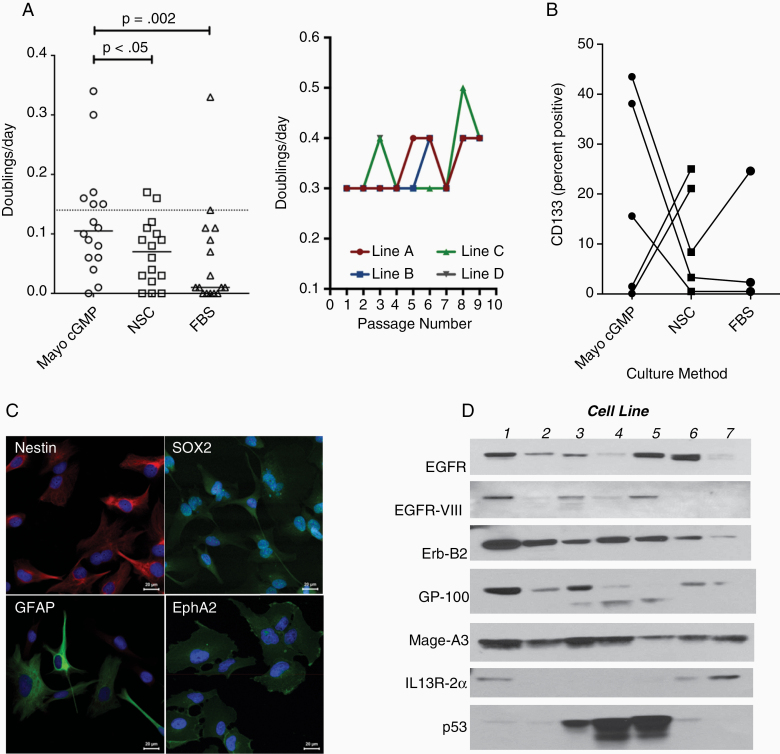
Novel method of cGMP tumor cell growth. Human glioblastoma cell lines were established either using FBS, NSC cultures or Mayo cGMP condition. (A) Growth kinetics of 16 lines (doublings/day) in each condition. Dashed line: doubling cell count per week (~1.4 doublings/day). One line was split into 4 subcultured over 10 passages (doublings/day over time). (B) CD133 expression in 5 lines. (C) Confocal micrographs from a representative cell line showing nestin (red) and SOX2, GFAP, and EphA2 (green) expression. Blue = DAPI. (D) Western blot (7 lines) showing glioma-associated antigen expression.

Sixteen operative GBM specimens were brought into tissue culture. Mayo cGMP was more effective for establishing cultures (15/16; 94%) similar to NSC (13/16; 81%) but more effective than FBS (7/16; 44%; *P* = .03, *χ*^2^; Figure 1A). Similarly, GBM cultures in Mayo cGMP media grew faster (mean doublings/day = 0.12; mean doubling time = 8.3 days) than NSC (mean doublings/day = 0.07; mean doubling time = 14 days; *P* < .05) or FBS (mean doublings/day = 0.06; mean doubling time = 17 days; *P* < .002). Efficient doubling (defined as 1 doubling/week) occurred in 44% of Mayo cGMP cultures compared with 13% for both NSC and FBS. Finally, doubling times remained relatively constant among efficiently growing Mayo cGMP cultures up to 10 passages (suggesting an absence of additional mutations over time causing instability).

### Mayo cGMP Human GBM Cell Lines Express Stem-Like Markers and Tumor-Associated Antigens

Mayo cGMP human GBM cell lines generally expressed the putative glioma stem cell marker CD133^25,26^ more frequently than matched NSC or FBS cell lines, though with variability between individual matched cell lines (Figure 1B). Confocal immunostaining demonstrates immature glioneuronal marker expression (nestin and SOX2; Figure 1B) as well as mature glioneuronal markers (GFAP and the tumor-associated antigen ephrin A2). Western blot confirms the expression of multiple tumor-associated antigens, though with substantial variation between cell lines (Figure 1C).

### Mayo cGMP Human GBM Cell Lines Have Stable Karyotypic Abnormalities

**Figure 2.**
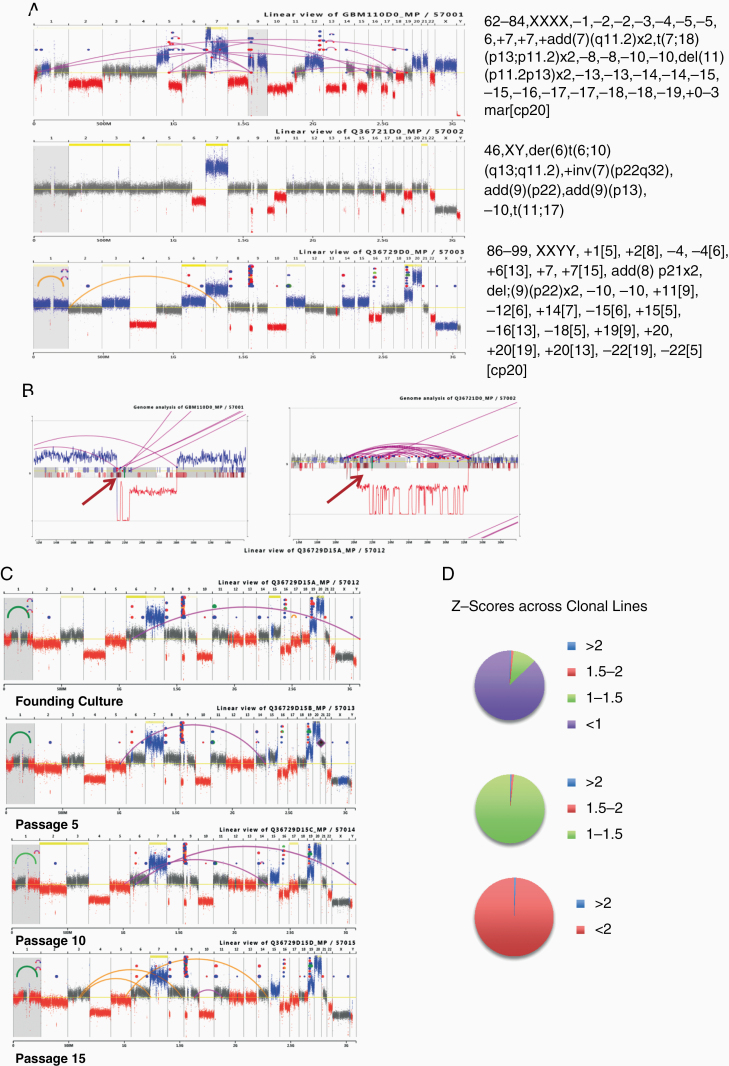
Stable GBM-associated cGMP tumor cell karyotypic abnormalities. (A) Mate-pair sequencing of 3 lines showing chromosome 7 gain, partial loss of 9, 10, and 22, and homozygous p16 deletion. (B) High-resolution p16. Homozygous deletion is indicated by an arrow. Chromothrypsis in Q36721 can be seen in right panel. (C) Long-term genetic stability. Four subclones of Q36279 were grown for up to 15 doublings. Samples were submitted for mate-pair sequencing and each line is shown. (D) *Z*-scores indicate very limited genetic drift during the course of expansion.

MPSeq was used to assess 3 representative Mayo cGMP human GBM cell lines. All 3 have distinct structural variance (Figure 2A). Each tumor predicts a tetraploid genome with genome doubling and additional gains/losses of chromosomes. Totals of 43, 86, and 54 junctions were detected in the initial clones for these 3 lines, respectively. The first 2 lines presented with significant numbers of inter-chromosomal translocations but all events in the third line were intra-chromosomal. Consistent karyotypic abnormalities (eg, gain of chromosome 7 and loss of chromosomes 10 and 22) characteristic of GBM^27,28^ were observed in all 3 lines (Figure 2A and B). Furthermore, despite the significant variation between the 3 tumors, each presented with chromothryptic events on chromosome 9p, with resulting homozygous deletion of *CDKN2A*. This is also characteristic of GBM (particularly its epitheloid variant).^29^ Complex rearrangements were common but, despite the common hit on chromosome 9, additional events were distinct across the genome (line 1, chormoplextic event 9q-5q and chromothryptic event 12q; line 2, chormoplextic events 9q-21 and 11-17-20; line 3, distinct chromothryptic events on 8p, 9q, 16, and 19). High resolution of the classic GBM p16 deletion for 2 of the cell lines is shown in Figure 2B (the start of which is indicated by an arrow), with a corresponding chromothryptic event seen in the right panel.

The third cell line is very stable through propagation to 15 passages, and no new copy number changes in chromosomes are predicted (Figure 2C). In order to assess variance in potential subclonal populations, absolute *Z*-scores were calculated for variance in the number of supporting reads detected in 4 subclones from different time points (passages 0, 5, 10, and 15) for the 54 commonly detected events (Figure 2D). Supporting reads were initially corrected for the total number of fragments mapping across the sequenced samples. Absolute *Z*-scores varying greater than 2 indicate significant variance from the norm. Just 0.7% (2 of 270 measurements of variance [54 junctions × 5 samples]) and 2.8% (20 of 702 measurements of variance [54 junctions × 13 samples]) presented with a significant absolute *Z*-scores greater than 2 for mean values from the 4 subclones or all samples individually, respectively. The majority of supporting reads detected (98.1% or 88.0%) had absolute *Z*-scores less than 1.5 for propagated lines or all samples, respectively. These results indicate only minor deviance from the norm of subclonal populations within the clones, suggesting very stable clonal populations.

### Novel Culture Methods Are Required to Generate Mature DCs From GBM Patient’s CD14+ Monocytes

**Figure 3.**
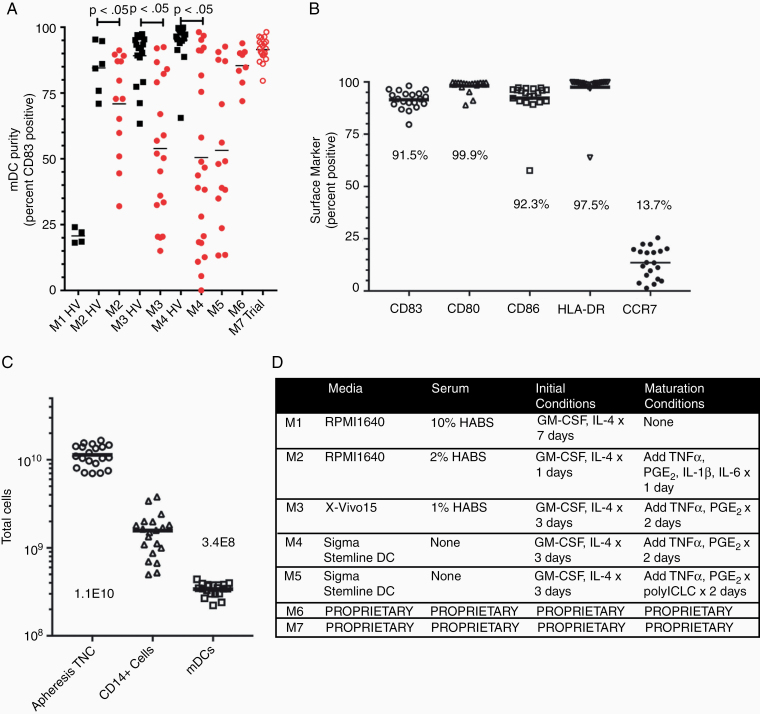
Optimized generation of mature dendritic cells for clinical-grade production. (A) Efficient mature DC (CD83+) generation. Various culture conditions (M1–M7) were tested in healthy volunteers (HV; black) or GBM patients (red). (B) M7 was used in clinical-scale cGMP manufacturing of 22 newly diagnosed GBM patients for enrollment in a pilot vaccine trial. Five markers (CD83, CD80, CD86, HLA-DR, and CCR7) describing the purity and potency of the cells are indicated. The mean percent positive for each marker is shown. Note: same patients as M7 in 3A. (C) Cell number after apheresis, isolation of CD14+ cells, and mature dendritic cell (mDC) culture with M7. (D) Culture conditions for DC culture M1–M7.

Peripheral blood CD14+ monocytes from healthy volunteers and GBM patients were cultured with a variety of DC culture techniques and frequency of mature CD83+ DCs was determined (Figure 3A). Note that this analysis was over a relatively long period of time as new methods were successively developed and then discarded due to poor yield with GBM patients. Thus, the patients in each condition varied in number and clinical scenario (newly diagnosed vs recurrent), though all patients were off therapy at the time of processing. A technique analogous to that used to generate DCs without a dedicated maturation step in many GBM vaccine clinical trials^2,6,30,31^ (M1) had low levels of CD83+ cells, as expected (20.7% ± 2.9%). Another technique incorporating a maturation step analogous to a different but commonly used technique in GBM DC vaccine clinical trials^32–35^ (M2) efficiently generated CD83+ mature DCs from healthy volunteer monocytes (84.5% ± 9.8%) but not from GBM patients (70.9% ± 19.3%; *P* < .05). We then empirically assessed 5 additional variations on culture techniques to generate mature DCs from GBM patient and/or healthy volunteer monocytes before achieving success with a technique (M6) that generated mature CD83+ DCs at frequencies (85.4% ± 7.2%) that were no longer significantly different from mature CD83+ DC frequency from healthy volunteer monocytes using optimized techniques. Finally, further minor variation to this technique (M7) resulted in an even higher frequency of CD83+ DCs (91.5% ± 4.4%) from GBM patients’ monocytes enrolled in a pilot clinical trial.

To confirm the generation of mature DCs in a more controlled patient population, we then used our optimized technique to generate mature DCs from 22 recently diagnosed adult GBM patients who had already undergone surgical resection as well as radiation with concurrent temozolomide but had not yet initiated adjuvant temozolomide (Figure 3B). These patients underwent apheresis to acquire peripheral blood mononuclear cells as part of potential enrollment in a clinical trial of DC vaccination in newly diagnosed GBM, the full results of which will be reported separately. In keeping with being mature DCs, expression of CD80 (98.2% ± 3.0%), CD86 (92.3% ± 8.9%), and HLA-DR (97.5% ± 8.0%) was also high and CCR7 was positive for a portion of the cells (13.6% ± 7.4%). Finally, the overall DC yield was good (3.4 × 10^8^ ± 5.5 × 10^7^ cells; Figure 3C). This represents an efficiency of about 21% mature DC generation from the purified population of CD14+ cells (1.6 × 10^9^ ± 8.8 × 10^8^). This manufacturing protocol yields enough mature DCs to produce an average of more than 13 doses of vaccines (approximately 2.5 × 10^7^ cells/dose) from single apheresis in a GBM patient.

### T Cells Stimulated With Autologous Mature DCs Pulsed With Allogeneic GBM Cell Line Lysate Kill HLA-A2-Matched GBM Cells

**Figure 4.**
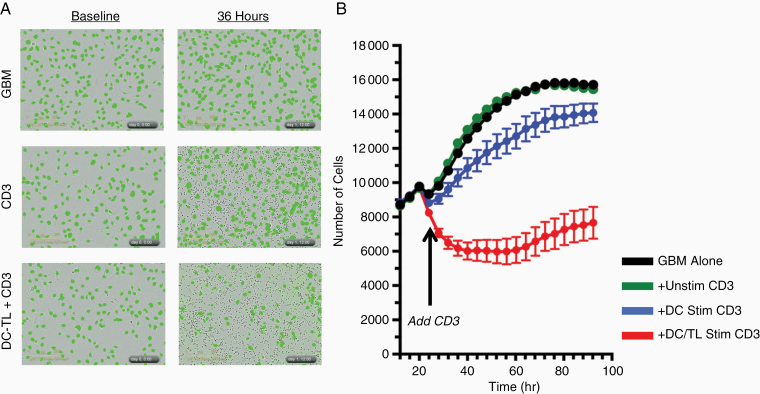
Autologous dendritic cell/allogeneic tumor lysate directed killing. Representative fluorescent micrographs (A) and time plots (B) showing reduced glioblastoma cells (green) after adding HLA-A2-matched CD3+ T cells stimulated with autologous DC pulsed with tumor lysate (+DC/TL Stim CD3; red line) compared to CD3 stimulated with autologous DC without tumor lysate (+DC Stim CD3; blue line), unstimulated CD3 (+Unstim CD3; green line) or controls (GBM alone; black line). Mean ± SD; *n* = 4. Representative results from 1 of 2 separate experiments are shown.

Using mature DCs generated with our M7 protocol, our fluorescent killing assay shows that autologous T cells stimulated with DCs pulsed with Mayo cGMP GBM cell line lysate robustly kill HLA-A2-matched GBM cells (Figure 4A). No killing is seen with unstimulated T cells. T cells stimulated with mature but naïve (ie, not pulsed with TL) DCs show very mild tumor killing, but much less than those stimulated with TL-pulsed DCs, suggesting some degree of non-antigen-specific T-cell stimulation (Figure 4B).

## Discussion

Although DC vaccines for GBM have been in clinical trials for nearly 2 decades^36^ and have shown considerable promise,^2,6,30–35^ they have yet to be FDA approved or widely adopted in clinical practice. There are multiple potential reasons for this, but issues related to antigen choice, DC potency, and manufacturing feasibility may be important contributors. In this article, we have outlined a novel allogeneic glioma cell/autologous DC platform that addresses these issues.

First, human GBM cell lines established with our platelet lysate-based Mayo cGMP protocol have many features that could make them ideal antigen sources for DC vaccines (Figure 1). They grow quickly—faster than matched human GBM cells grown in either neural stem cell conditions or standard serum-containing conditions. This underscores the feasibility of using these cell lines as an antigen source. They appear to have a mixture of stem-like and more differentiated markers. Expression of the putative stem-like marker CD133^37^ is actually higher in these cell lines than in matched lines established with stem cell media. However, they grow in monolayers and express a spectrum of common glioma-associated antigens and mature glioneuronal markers more typically associated with differentiated glioma cells, suggesting that they may represent a mix of these phenotypes. This is particularly attractive as an antigen source for glioma vaccines which would ideally target both stem-like and differentiated cells. Finally, the established presence of these common glioma-associated antigens in a renewable antigen source could allow antigen-specific response testing in the context of clinical trials that cannot be accomplished in patient-derived bulk antigen vaccines.^6,7^

The cell lines established with our Mayo cGMP protocol appear stable over time, highlighting their utility as a renewable bulk antigen source. Although the cell lines grow quickly, their growth does not appear to accelerate much over time up to 15 passages (Figure 1A). Furthermore, MPseq over time also shows stability (Figure 2). However, it should be noted that a subgroup of events not detected in every specimen consisted of events with lower numbers of supporting reads, which fall below the reporting threshold for MPseq data. Hence, the majority were detectable to the level of at least 1 supporting read in the majority of specimens. Increasing the sequencing depth would be expected to report these events at higher confidence. Therefore, while whole-genome-based sequencing techniques may not provide the greatest sensitivity for detecting new variant clones in a passage specimen, they are well-suited for predicting overall clonal stability. New variations would be expected to initially occur in 1 single cell, which would take time to preferentially expand to a level which can be detected by a lower sensitivity whole-genome-based technique. The number of supporting reads crossing a breakpoint junction provides clues as to the frequency of such an event in a cell line as a whole. Ideally in a pure 100% tumor cell line, the number of supporting reads of a single occurring junction (ie, no copy gain) should equal half the allelic bridged coverage (at 50× genome coverage, the average event supporting read coverage should be approximately 25). However, significant variance of these values from the ideal is frequently observed. Tumor heterogeneity results in lower numbers of supporting reads for lower represented clones. Repetitive genome regions challenge MPseq mapping because a single sequence could be mapped to multiple sites. MPseq is often able to span over these repetitive regions, but the total number of mapping events can be reduced. In total 90 somatic junctions (3 or more supporting reads) were detected in one cell line in the founding day 0 colony and 12 additional clones from 5, 10, and 15 passages of 4 independent clonal lines. Of these 90 junctions, 54 (60%) were detected in every clone. The remaining 36 events presented with lower supporting reads across clones indicative of potential subclonal populations in the cell lines or events lying in repetitive regions of the genome difficult to assess by mate pair. However, in total, 83 of the 90 events (92.2%) presented evidence in at least 1 clone from the 4 independent clonal lines. Just 2 events, both with 3 supporting reads, presented evidence in just 1 clone, each within early-passage 5 clones. Taken together, this method appears to produce primary tumor lines at high frequency, with stable genetics, under cGMP conditions suitable for therapeutic use.

DC culture techniques that do not incorporate maturation steps (eg, technique M1) do not result in significant numbers of CD83+ cells even with healthy donor monocytes (Figure 3A). Standard culture techniques for generating mature DCs (eg, technique M2) produce many CD83+ cells when performed with healthy donors’ monocytes but not when using GBM patients’ monocytes. We suspect that this reflects the high frequency of immunosuppressive monocytic cells such as myeloid-derived suppressor cells within GBM patients’ CD14+ monocytes.^16,17^ Through empiric modification, we were able to develop a culture method that results in high levels of CD83+ mature DCs (technique M7). These cells express high levels of other DC markers including HLA-DR, CD80, and CD86 (Figure 3B). The efficiency of this method to generate mature CD83+ DCs from CD14+ precursors was about as expected, that is, on average 3.4 × 10^8^ per patient (Figure 3C). This would correspond to more than 13 doses of vaccine at 2.5 × 10^7^ cells per dose. Thus, this is a highly feasible method for vaccine manufacture.

Putting this all together, T cells stimulated with autologous DCs generated with our optimized technique (M7) and pulsed with our allogeneic GBM cell lysate show potent killing ability against HLA-A2-matched GBM cell lines (Figure 4). This is much more marked than the moderate increase in GBM cell killing by T cells stimulated with DCs that were not pulsed with GBM cell line lysate, though the presence of this killing suggests some degree of non-antigen-specific T-cell activation (Figure 4B). No GBM cell killing at all was seen for unstimulated T cells.

Thus, we have outlined a novel method for manufacturing DC vaccines for GBM that addresses several deficiencies in standard approaches. Our Mayo cGMP protocol generates stable human GBM cell lines that represent a stable and self-renewing source of GBM-associated antigens. This obviates the need for tumor tissue derived from individual patients for vaccine manufacture and would allow antigen-specific response testing in the context of clinical trials. Our DC culture technique is a highly efficient and feasible method for generating large numbers of mature DCs from CD14+ monocytes derived from a single apheresis in GBM patients. Finally, the combination of these DCs pulsed with our GBM cell line lysate stimulates T cells that potently kill HLA-A2-matched GBM cells. This vaccine platform is, therefore, both highly feasible for manufacture and highly potent. It is necessary to test the safety and feasibility of this approach in GBM patients as a next step. Therefore, we have initiated clinical trials of this vaccine platform in both newly diagnosed (in combination with temozolomide chemotherapy; NCT01957956) and recurrent GBM (as a single agent; NCT03360708) patients.

## Supplementary Material

vdaa105_suppl_Supplementary_Table_1

## Notes

**Conflict of interest statement**. M.P.G. and A.B.D. are inventors of technology related to establishing cell lines used in this article. The technology has been licensed to Mill Creek Life Sciences; Mayo Clinic and the inventors have contractual rights to receive royalties from the licensing of this technology. In addition, Mayo Clinic and A.B.D. hold equity in the company to which the technology is licensed. This conflict is managed according to policies and procedures regarding conflict of interest at Mayo Clinic.

## Notes

**Authorship statement**. Study conception/design: I.F.P. and A.B.D. Data acquisition: all authors. Data analysis/interpretation: I.F.P., M.P.G., T.E.P., S.J.M., G.V., and A.B.D. Manuscript drafting/editing/approval: all authors.
