# Supplementary material for: Novel strategy for manufacturing autologous dendritic cell/allogeneic tumor lysate vaccines for glioblastoma
Source: Neurooncol Adv. 2020 Aug 26;2(1):vdaa105. doi: 10.1093/noajnl/vdaa105 (PMC7592424; doi:10.1093/noajnl/vdaa105)
Supplement: vdaa105_suppl_Supplementary_Table_1 [file vdaa105_suppl_supplementary_table_1.docx]

| Target | Source | Clone/ Cat # |
| --- | --- | --- |
| CD80 | Coulter | MAB104 / IM1853U |
| CD83 | Coulter | HB15a / IM2410U |
| CD86 | Coulter | HA5.2B7 / IM2729U |
| CD133 | Coulter | W6b3c1 / C15190 |
| HLA-DR | Coulter | Immu-357 / B20024 |
| Nestin | Millipore | Clone 10C2/ #MAB5326 |
| SOX2 | Santa Cruz | #sc-17320 |
| GFAP | Millipore | Clone GA5 / #MAB360 |
| EphA2 | Cell Signaling | Clone D4A2 / #6997s |
| EGFR | Abcam | Clone ICR10 / #ab231 |
| EGFRviii | Zymed | #32-6400 |
| erb-B2 | Cell Signaling | Clone D8F12/ #4290s |
| gp100 | Santa Cruz | #sc-15010 |
| MAGE-A3 | Abcam | #ab38496 |
| IL13Rα2 | Santa Cruz | Clone 2K8 / #sc-134363 |
| p53 | Santa Cruz | Clone DO-1/#sc-126 |
